# Application of a pre-emptive question and answer platform to improve the level of satisfaction during family meetings in general medical wards

**DOI:** 10.1186/s12913-022-07929-z

**Published:** 2022-04-14

**Authors:** Wen-Chun Yu, Chun-Ta Huang, Wang-Huei Sheng

**Affiliations:** 1 Department of Nursing, National Taiwan University Hospital, Taipei, Taiwan; 2 Department of Internal Medicine, National Taiwan University Hospital, No. 7, Chung-Shan South Road, Taipei, Taiwan; 3 Graduate Institute of Clinical Medicine, National Taiwan University, Taipei, Taiwan

**Keywords:** Communication, Family, Quality of care, Questionnaire, Satisfaction

## Abstract

**Background:**

A successful family meeting is key to family-centered care and may foster communication and improve the level of satisfaction of the family in terms of communication. In this study, we evaluated whether a proactive communication channel could improve the level of satisfaction of family members following a family meeting in a general medical ward setting.

**Methods:**

We conducted a pre- and post-study to compare the level of satisfaction of a family with a family meeting before (*N* = 39) and after (*N* = 29) intervention in two general medical wards of a tertiary-care referral center. The intervention included a pre-emptive question and answer platform and a written response to family-raised queries in addition to a regular setting. Following each family meeting, family members were requested to fill a 10-item survey assessing their levels of satisfaction.

**Results:**

The characteristics of the family members in terms of demographics, education levels, and previous experiences with family meetings in the pre- and post-intervention groups were similar. The scores in all the items that indicated the level of satisfaction significantly improved after intervention. The overall score for satisfaction increased from 85 (interquartile range, 80–95) to 98 (interquartile range, 93–100; *P* < 0.001).

**Conclusions:**

Compared with conventional practice, the inclusion of a proactive communication platform along with a written response to raised queries as a part of family meetings improved the satisfaction levels of the family in terms of the content and process of the meeting in the general ward setting. Further studies are needed to delineate the optimal timing and use of such a communication modality.

**Supplementary Information:**

The online version contains supplementary material available at 10.1186/s12913-022-07929-z.

## Introduction

A family meeting, in a narrow sense, refers to a discussion between a physician and family members about certain patient-related topics and is a useful tool to facilitate communication between healthcare providers and the families of hospitalized patients. Good communication is associated with improved levels of satisfaction, trust in physicians, decision-making, and psychological health of the family members [1–5]. A number of protocols have been established as a guide for conducting family meetings, particularly in the settings of critical care, palliative care, and pediatrics, to foster a successful family meeting [6–9]. However, little is known on the role and structure of a family meeting in the general medical floor, outside these settings. Recently, Gambhir et al*.* reported that a scheduled and structured family meeting for general medicine patients led to an improved understanding among the patients regarding their care and level of satisfaction [2]. Their study findings support the importance of involving the patients and family members in the care process by conducting a successful family meeting in the general ward similar to that conducted in the intensive care unit [6].

Ideally, family members should be provided sufficient time and opportunities to ask questions, raise issues, express concerns, and confront unfavorable emotions with the help of the patient and a compassionate healthcare team in a family meeting [10, 11]. Allowing family members more time to speak during the meeting may improve the level of satisfaction of the family in terms of communication and conflicts with physicians [12]. Some experts suggest that, in a family meeting, family members should be offered at least the same amount of time that physicians have to speak as an important part of family-centered care [8, 13]. Nonetheless, nowadays, a rushing and pressing healthcare environment may drive families away from such opportunities [14]. Therefore, to conduct an efficient and successful family meeting, a new approach to elicit the needs and views from the family members is urgently warranted.

In this regard, our team proposed and built a communication channel for family members as a platform for pre-emptive questions and answers before conducting a formal family meeting. In the present study, we aimed to investigate whether incorporating this platform into a regular family meeting improved the level of satisfaction of family members with regard to the content and process of the meeting in a general floor setting.

## Methods

### Study settings and population

The study was conducted as a pre- and post-intervention design from September 2020 to April 2021. The study population consisted of family members of patients who had been hospitalized in the two general medical wards of the National Taiwan University Hospital (NTUH), a large tertiary-care referral medical center in Northern Taiwan. These two medical wards were specifically dedicated to accommodating patients from the emergency care unit. Patients with any diagnosis were eligible. We included family members with: a) an age ≥ 20 years and b) adequate knowledge of the Chinese language. The study was conducted in line with the principles stated in the Declaration of Helsinki, and the study protocol was exempted from ethics review by the Research Ethics Committee of the NTUH.

### Satisfaction survey

The survey consisted of questions regarding demography and family members’ prior experience with a family meeting along with a 10-item questionnaire assessing the level of satisfaction. The questionnaire was aimed at rating participants’ level of satisfaction with family meetings in terms of communication, shared decision-making, understanding, and respect. The 10 items were adapted from the institutional satisfaction survey of the NTUH and modified to suit the scenario. The family members could indicate on a five-point Likert scale how they agreed or disagreed with the statement. The five-point Likert scale comprised the following responses: strongly agree, agree, neither agree nor disagree, disagree, and strongly disagree, and the corresponding scores of 100, 75, 50, 25, and 0, respectively, were assigned to the responses for statistical analysis.

The first draft of the questionnaire was created by the study team and six reviewers reviewed the relevance and wording of each item using a 4-point rating scale. The panel consisted of six experts from various academic and working backgrounds, including one MD–PhD in Health Policy and Management, one MD–PhD in Epidemiology and Preventive Medicine, one MD with expertise in multidisciplinary patient care, one RN–PhD in Psychiatric Epidemiology and Mental Health, one RN–PhD with expertise in healthcare quality and patient safety, and one RN–MS with expertise in patient referral management.

For the evaluation of the relevance, the responses on the 4-point scale were as follows: 1 = not relevant; 2 = somewhat relevant; 3 = quite relevant; and 4 = very relevant [15–17]. The content validity of each item was determined using the content validity index (CVI), calculated as the number of panel members providing a rating of 3 or 4 divided by the total number of the panel members [16, 17]. The clarity of the wording was judged based on the following responses: 1 = not clear; 2 = somewhat clear; 3 = quite clear; and 4 = highly clear [18]. The face validity of the questionnaire items was determined using the face validity index (FVI), calculated as the proportion of panel members giving an item a clarity rating of 3 or 4 [18]. The acceptable CVI and FVI at the item-level were both set at a minimum of 0.80 [16–18]. The CVI scores assigned by the panel members were 0.83 for questionnaire items 6 and 8, and 1.00 for the other items. The FVI scores rated by the panel members were 0.83 for items 2, 3, and 7, and 1.00 for the others. The second draft of the questionnaire was created after incorporating minor revisions in terms of language based on the panel members’ opinions.

Subsequently, the questionnaire was pilot-tested to evaluate its internal consistency and inter-rater reliability. We included 30 sessions of family meetings at this stage. For each family meeting, two family members were requested to fill the questionnaire. The questionnaire showed good internal consistency (Cronbach’s α = 0.797). We also calculated the inter-rater reliability using the intraclass correlation coefficient (ICC) and we found a strong agreement between family members (ICC = 0.763). The questionnaire was finalized after pilot-testing (Additional file 1).

### Intervention

The intervention consisted of two parts. The first was a website, the communication channel, containing an online blank form through which family members could submit their questions or comments on patient care. On the website, a few sentences explained the purpose of the study and encouraged family members to raise patient-associated issues. A QR code was generated to aid in easily accessing the website. The second was a written point-by-point response to the queries that family members posted on the website. The responses were drafted by the resident or nurse practitioner and finalized by the attending physician. The study procedures are narrated in detail below.

Before the intervention, family members raised their questions and issues in a verbal or written format as per their needs prior to conducting a family meeting. The attending healthcare team usually answered the questions or discussed the issues verbally in the meeting. During the post-intervention period, a pre-emptive question and answer platform was used. At least 24 h before convening the family meeting, family members were provided with the QR code to connect to the website on which they left questions and issues that they wanted addressed. The healthcare team prepared the answers to the questions or the information addressing the issues in a written format in advance. At the beginning of the family meeting, family members were provided with the documents and their pre-emptive questions and issues were clarified after introducing family members and healthcare providers each other. Later, the family meeting proceeded in the conventional manner. Following each of the family meetings, the family members were requested to fill the surveys on the family members’ levels of satisfaction.

### Sample size estimation

Assuming a variance of approximately 64 for the overall score for satisfaction in each group, we included at least 40 participants in each group to detect a difference of at least five points in the median overall satisfaction score between the pre- and post-intervention groups with a power of 0.80 and an α of 0.05. The predicted difference between the groups was estimated based on the pilot-testing results.

### Statistics

Data were reported as numbers (percentages) or medians (interquartile ranges) as appropriate. To examine whether there were any differences between the levels of satisfaction of the family based on the questionnaire items before and after the intervention, the Mann–Whitney U test was used. Comparisons between the family’s baseline characteristics were performed using the χ^2^ test. Cronbach’s α was adopted to examine internal consistency among the items. All tests were two-tailed and a *P* value of < 0.05 was regarded as statistically significant. All statistical analyses were performed using the Statistical Package for Social Sciences (SPSS, version 22.0, SPSS Inc., Chicago, IL).

## Results

**Fig. 1:**
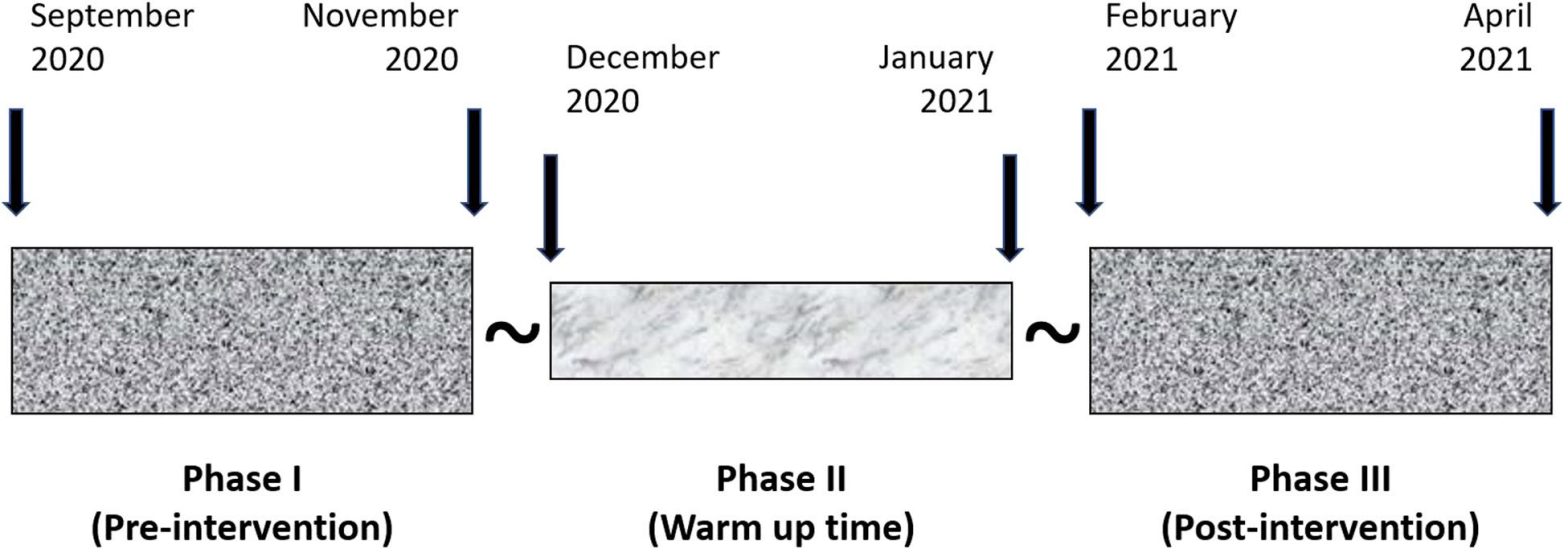
Study timeline. A before-and-after study was conducted in three phases. Questionnaires for grading the level of satisfaction were administered in phases I (pre-intervention) and III (post-intervention) of the study but not in phase II (warm-up time)

Figure 1 shows the study timeline. During the study period, 41 and 30 questionnaires were distributed in the pre- and post-intervention phases, with a response rate of 95% (*N* = 39) and 97% (*N* = 29), respectively. The post-intervention phase was prematurely halted because of the escalation of COVID-19 restrictions in Taiwan in May 2021. Table 1 shows the characteristics of the study participant. The majority of family members present at the meeting, i.e., 79% and 76%, respectively, in the pre- and post-intervention phases, were close family members (spouse, child, and parent), and the rest were second- or third-degree relatives. Only three patients each during the pre- and post-intervention periods were present for the meeting. We found no statistically significant differences between the study groups with regards to demographics, education levels, and previous experiences with family meetings.

**Table 1:** Characteristics of the study participants

Variables	Pre-intervention	Post-intervention	*P* value
Participant No	39	29	
Age groups			
20–39	8 (21)	8 (28)	0.723
40–59	21 (54)	13 (45)	
60–79	10 (26)	8 (28)	
Gender			
Female	19 (51)	16 (45)	0.598
Male	20 (49)	13 (55)	
Level of education			
High school or lower	20 (51)	16 (55)	0.463
College	17 (44)	13 (45)	
Advanced	2 (5)	0 (0)	
Family member’s relationship to the patient			
Spouse	9 (23)	6 (21)	0.951
Parent	3 (8)	2 (7)	
Child	19 (49)	14 (48)	
Sibling	2 (5)	3 (10)	
Others	6 (15)	4 (14)	
Earlier experience of family meetings			
Present	34 (87)	22 (76)	0.226
Absent	5 (13)	7 (24)	

In terms of patient characteristics, the median age was 82 (range 61–97) years and most (52%) of the patients were males. The principal diagnosis at admission for these patients was infectious diseases (77%), including pneumonia, urinary tract infection, and intra-abdominal infection. Throughout the study, family meetings were held on an as-needed basis, primarily to discuss patient situations and for informed decision-making.

During the pre-intervention period, the median satisfaction score of the majority of questionnaire items was 75 (Table 2), and the overall level of satisfaction of the family members was 85 (80–95). The internal consistency for all items assessed using the Cronbach’s α was 0.977. After the intervention, the scores of all the items assessing the satisfaction levels of the family significantly improved, with a median score of 100. The overall satisfaction score also significantly increased to 98 (93–100; *P* < 0.001). The internal consistency across questionnaire items remained stable and extremely high over this study period (Cronbach’s α = 0.921).

**Table 2:** Questionnaire items and scores, pre- and post-intervention

Items	Pre-intervention	Post-intervention	*P* value
1. The staff can keep me well informed on what has happened to my family member	75 (50–100)	100 (75–100)	< 0.001
2. The staff can treat me well with respect and autonomy	75 (50–100)	100 (75–100)	< 0.001
3. All my concerns can be adequately addressed and questions to be answered	75 (50–100)	100 (75–100)	< 0.001
4. I can feel supported and confident in the decision making process	75 (50–100)	100 (75–100)	< 0.001
5. I can fully realize my family member’s future health condition	75 (50–100)	100 (75–100)	< 0.001
6. I can better understand how to take care of my family member	75 (50–75)	100 (75–100)	< 0.001
7. I can be more comfortable with discharge planning and disposition	75 (50–75)	100 (75–100)	< 0.001
8. I can learn more about how to obtain available medical facilities and resources	75 (50–75)	100 (75–100)	< 0.001
9. I am more satisfied with the content of this discussion than my previous experience	50 (50–75)	100 (100–100)	< 0.001
10. Please rate the overall satisfaction of your experience with the family meeting (0–100)	85 (80–95)	98 (93–100)	< 0.001

## Discussion

The optimal healthcare experienced by family members is commonly described as family-centered care [8]. A family meeting is an ideal venue to deliver such a type of care and is central to the patient’s care plan [19]. For critically-ill patients, proactive interventions, including intensive communication and provision of a family information leaflet, improved family comprehension, decreased the levels of anxiety and depression, and increased the level of satisfaction among family members [1, 20–22]. In our pre- and post-intervention study, we compared two family meeting formats, one using a proactive approach to communication and the other using the conventional approach to a family meeting in the general wards. The level of satisfaction in terms of the family meeting rated by family members was significantly improved among families that attended meetings that followed the proactive communication strategy, via pre-emptive questions and answers and a written response to the questions. Our findings suggest that some family needs are universal and should be proactively met across a variety of healthcare settings.

One of the advantages of pre-emptive questions and answers include the possibility of better fitting family needs during the family meeting. The communication platform encourages and allows family members to take time to think about the questions and issues that need to be addressed, and healthcare providers can and will prepare the answers and responses for these family-centered issues prior to the meeting. Moreover, the platform serves as a channel for listening to the family’s non-verbal voices, which is an important part of a successful and satisfactory family meeting [12, 13]. As more family needs can be exposed and met using the problem-solving process, it is not unexpected that family members were more satisfied with the meeting during the post-intervention period.

Another advantage of pre-emptive questions and answers is that the healthcare team shows their desire, enthusiasm, and compassion to help family members deal with a difficult time in their lives. A multicenter study conducted in Canada revealed that compassion shown to the family members was significantly associated with the overall level of satisfaction with patient care in the intensive care unit [23]. There was also an association between more empathic statements and higher levels of family satisfaction with communication in terms of life support-based decisions in the intensive care unit [24]. Though not in a verbal form, the pre-emptive question and answer platform helped in expressing concern regarding and sympathizing with the family members. Thus, they might have felt emotionally supported and provided high ratings for the level of satisfaction with the family meeting process.

In the post-intervention period, family members were provided with a written response to their pre-emptive questions and issues combined with a verbal elaboration. A randomized, controlled trial by Azoulay et al*.* revealed that the delivery of a family information leaflet as a part of communication improved comprehension without providing patient-specific information and good comprehension was associated with better levels of satisfaction in the ICU [20]. Another trial by the same group also found that a bereavement brochure provided to the family members along with conducting a family meeting decreased symptoms of anxiety and depression among family members of dying ICU patients [1]. The favorable outcome of ICU studies prompted us to incorporate a question and answer form into our communication intervention in the non-ICU setting. Our data suggest that written information specific to family needs may be helpful in improving the understanding and level of satisfaction in terms of the content of a family meeting.

In palliative care, a patient-centered family meeting is a new approach to listening to the patient’s voice and prioritizing and addressing the patient’s issues [25–27]. Active patient involvement may provide additional and valued opportunities for patients to express mutual concerns, deliver messages of comfort and appreciation, and prepare for death [27]. In this study, however, only few patients were present in the family meeting, and family members played a dominant role in the discussion. The different settings in which the family meeting was held may partly explain the discrepancy, but the major determinant might be the cultural difference. The principle of autonomy in Western society focuses on self-determination and promotes the value of individual independence, whereas the principle in East Asian society requires family-determination and upholds the value of harmonious dependence [28]. In this regard, the proportions of patients who were involved in the discussion on end-of-life issues in East Asian countries [29, 30] were far less than those in Western countries [31, 32]. Thus, it is not surprising that a similar finding was observed in our general floor setting.

Our study has a couple of limitations. First, we included a small number of participants, and this study represented a single-center experience with the application of a pre-emptive question and answer platform in the family meeting. Thus, the results may need to be validated in other institutional settings with a larger population. However, to the best of our knowledge, our study was a pioneer study to introduce a new communication channel between family members and healthcare providers for family meetings in the general ward setting, and the findings of our study should facilitate more studies in this field. Second, verbal and non-verbal communication between family members and healthcare providers can certainly occur outside the family meeting setting, which may impact family satisfaction in terms of the family meeting. Our analysis herein cannot specifically address such an important confounder. Nonetheless, randomized, controlled trials are subjected to these confounding effects.

## Conclusions

In summary, compared with conventional practice, including a pre-emptive question and answer platform with a written response to family-raised issues as part of a family meeting significantly improved the level of satisfaction of the family with the content of the meeting in a general ward setting. The findings reinforce the importance of a proactive approach to conducting a family meeting in the general ward as that in the critical and palliative care settings. Further larger-scale studies are needed to confirm our results and delineate the optimal timing and use of such a communication modality.

## Supplementary Information

**Additional file 1.** Questionnaire items.

## Data Availability

The datasets used and/or analyzed during the current study are available from the corresponding author on reasonable request.

## Abbreviations

CVI: Content validity index
FVI: Face validity index
ICC: Intraclass correlation coefficient
NTUH: National Taiwan University Hospital
