# Supplementary material for: Application of a pre-emptive question and answer platform to improve the level of satisfaction during family meetings in general medical wards
Source: BMC Health Serv Res. 2022 Apr 14;22:499. doi: 10.1186/s12913-022-07929-z (PMC9008296; doi:10.1186/s12913-022-07929-z)
Supplement: Supplementary file 1 — Additional file 1. Questionnaire items. [file 12913_2022_7929_MOESM1_ESM.docx]

**Additional file 1. Questionnaire items**

*Part 1. Demographic information*

□ 20-39 □ 40-59 □ 60-79 □ 80 or older

□ male □ female

□ high school or lower □ bachelor □ master or higher

□ yes □ no

□ spouse □ parent □ child □ sibling □ others __________

1. Age group
2. Gender
3. Education background
4. Any prior experience with a family meeting
5. Relationship to the patient

*Part 2. Satisfaction survey*

| Questions | Strongly Agree | Agree | Neither Agree nor Disagree | Disagree | Strongly Disagree |
| --- | --- | --- | --- | --- | --- |
|  | (100) | (75) | (50) | (25) | (0) |
| 1. The staff can keep me well informed on what has happened to my family member. |  |  |  |  |  |
| 1. The staff can treat me well with respect and autonomy. |  |  |  |  |  |
| 1. All my concerns can be adequately addressed and questions to be answered. |  |  |  |  |  |
| 1. I can feel supported and confident in the decision-making process. |  |  |  |  |  |
| 1. I can fully realize my family member’s future health condition. |  |  |  |  |  |
| 1. I can better understand how to take care of my family member. |  |  |  |  |  |
| 1. I can be more comfortable with discharge planning and disposition. |  |  |  |  |  |
| 1. I can learn more about how to obtain available medical facilities and resources. |  |  |  |  |  |
| 1. I am more satisfied with the content of this discussion than my previous experience. |  |  |  |  |  |
| 1. Please rate the overall satisfaction of your experience with the family meeting (0-100). |  |  |  |  |  |
